# Association Between Dietary Pattern and Serum C-Reactive Protein in Japanese Men and Women

**DOI:** 10.2188/jea.JE20100110

**Published:** 2011-03-05

**Authors:** Hinako Nanri, Kazuyo Nakamura, Megumi Hara, Yasuki Higaki, Takeshi Imaizumi, Naoto Taguchi, Tatsuhiko Sakamoto, Mikako Horita, Koichi Shinchi, Keitaro Tanaka

**Affiliations:** 1 Department of Preventive Medicine, Faculty of Medicine, Saga University, Saga, Japan; 2 Laboratory of Exercise Physiology, Faculty of Sports and Health Science, Fukuoka University, Fukuoka, Japan; 3 Fukuoka Prefectural Government, Asakura Health Welfare Environment Office, Asakura, Japan; 4 Division of International Health and Nursing, Faculty of Medicine, Saga University, Saga, Japan

**Keywords:** dietary pattern, C-reactive protein, inflammation, factor analysis

## Abstract

**Background:**

Dietary pattern may influence the risks of cardiovascular disease, atherosclerosis, type 2 diabetes, and metabolic syndrome through its effects on inflammation. We evaluated the association between dietary pattern and serum high-sensitivity C-reactive protein (hs-CRP) in a Japanese population.

**Methods:**

In this cross-sectional analysis, we used baseline data from 3905 men and 5640 women (age 40–69 years) who participated in a population-based cohort study between November 2005 and December 2007. Participants with possible inflammation-related diseases, current analgesic use, high hs-CRP levels (≥3000 ng/mL) or extreme dietary energy intake were excluded. We used 46 items from a validated short food frequency questionnaire and examined major dietary patterns by factor analysis.

**Results:**

We identified 5 dietary patterns: healthy (high in vegetables and fruit), Western (high in meat and fried foods), seafood (high in shellfish, squid, fish, etc.), bread (high in bread and low in rice), and dessert (high in confections and fruit). After adjustment for age, alcohol use, smoking, physical activity, and body mass index, hs-CRP levels in men were inversely associated with the healthy, bread, and dessert patterns (*P*-trend: 0.01, 0.06, and <0.01, respectively) and positively associated with the seafood pattern (*P*-trend = 0.02). In women, hs-CRP levels were inversely associated with the healthy pattern (*P*-trend = 0.06) and positively associated with the Western pattern (*P*-trend = 0.06).

**Conclusions:**

The healthy dietary pattern may be associated with suppressed inflammation in Japanese men and women, independently of body mass index and other factors. The sex-specific associations of hs-CRP with other dietary patterns (eg, the seafood pattern) require further study.

## INTRODUCTION

Chronic inflammation is independently and directly associated with cardiovascular disease, atherosclerosis, type 2 diabetes, and metabolic syndrome.^1^^–^^5^ C-reactive protein (CRP) is an acute-phase protein involved in inflammation. It is secreted primarily by the liver after its synthesis is triggered by pro-inflammatory cytokines such as interleukin-6, which is thought to be released by adipose tissue.^2^ Current evidence suggests that elevated blood levels of CRP are associated with older age, obesity, smoking, no or heavy alcohol intake, and a lower level of physical activity.^6^^–^^10^ Intakes of some nutrients or foods (eg, n-3 polyunsaturated fatty acids [n-3 PUFAs], fiber, vegetables, fruit, and fish) have also been reported to be associated with serum CRP concentration.^11^^–^^14^

People typically consume meals consisting of a variety of foods, with complex combinations of nutrients, rather than isolated nutrients. The traditional approach of studying single nutrients or foods may therefore be limited in terms of 1) possible interactions and high intercorrelations between various food components, which may make it difficult to evaluate the overall or independent effects of different nutrients or foods, 2) possibly small and thus undetectable effects of a single nutrient, and 3) the issue of multiple comparison.^15^^,^^16^ Accordingly, dietary pattern analysis, which examines the effects of overall diet, has emerged as an alternative and complementary approach. The association between dietary patterns and serum CRP has been explored in several studies,^17^^–^^22^ but the results have been inconsistent.

An association between a healthy, or prudent, dietary pattern (ie, a diet with higher intakes of vegetables, fruit, and fish) and decreased levels of serum CRP has been reported in some studies,^18^^,^^20^^,^^21^ but not in others.^17^^,^^19^^,^^22^ In addition, several studies have demonstrated that a Western, or high fat and processed meat, pattern (ie, higher intakes of red meat, refined grains, processed meat, and high-fat dairy products) is related to elevated serum CRP,^17^^–^^20^ although some studies did not corroborate this finding.^21^^,^^22^ To our knowledge, there has been only 1 cross-sectional study of dietary patterns and CRP in Japan. In that study, a healthy dietary pattern was strongly associated with lower serum CRP, and a seafood pattern was possibly related to higher CRP.^21^ The objective of the present study was to evaluate more comprehensively the association between dietary pattern and serum CRP in a Japanese population.

## METHODS

### Subjects

In November 2005, we initiated a population-based prospective cohort study in Saga Prefecture, in northern Kyusyu island, as part of the Japan Multi-Institutional Collaborative Cohort Study (J-MICC Study), which aims to obtain fundamental data on the prevention and genetics of lifestyle-related diseases, particularly cancer.^23^ The subjects and methods of our cohort study (designated the Saga J-MICC Study) are described in detail elsewhere.^24^ Eligible subjects in the Saga study were all residents of the area corresponding to the former city of Saga (in the center of the current city of Saga) and were between the ages of 40 and 69 years at the time of the baseline survey for the Saga J-MICC Study. The former city of Saga was administratively consolidated with 4 adjacent municipalities on 1 October 2005. Candidates were enrolled by using the name, sex, date of birth, and address of all residents on the resident register in the city office. A total of 61 447 residents were invited by mail to participate in the baseline survey, which was administered between 1 November 2005 and 22 December 2007 in public halls and other available venues near the residential areas of participants. In total, 12 078 subjects (participation rate =19.7%; 5082 men and 6996 women) completed the baseline survey. Written informed consent was obtained from all participants, and the study protocol was approved by the Ethics Committees at both the Saga University Faculty of Medicine and the Nagoya University Graduate School of Medicine.

### Questionnaire survey and measurements

A self-administered questionnaire that included questions on alcohol consumption, smoking, and dietary habits, as well as current medication and past disease history, was sent to participants beforehand. They were instructed to bring their completed questionnaires to the survey location. On the day of the survey, a research nurse or nutritionist asked participants about any missing or inconsistent answers. Height and weight were measured to the nearest 0.1 cm and 0.1 kg, respectively. Venous blood was drawn from each participant, and serum, plasma, and buffy coat were separated within 3 hours and stored at −80°C until tested. Subjects were instructed to wear a single-axis accelerometer (Kenz Lifecorder EX; Suzuken Co. Ltd, Nagoya, Japan) on either side of the hip, except when sleeping or bathing, for 10 days after the baseline survey.

We assessed consumption of 6 types of alcoholic beverages: *sake*, *shochu* (a Japanese distilled beverage), *chuhai* (*shochu* plus mixer), beer, whiskey, and wine. The categories of consumption frequency were: almost none, 1 to 3 days/month, 1 to 2 days/week, 3 to 4 days/week, 5 to 6 days/week, and daily, and the amount of each beverage consumed per day was reported. Total ethanol consumption per day for current drinkers was estimated based on beverage-specific ethanol concentrations. As for smoking habits, subjects were first asked about current smoking status (and cessation time for former smokers). Current and former smokers reported their usual cigarette consumption (cigarettes/day) and the age at which they started smoking. Body mass index (BMI) was calculated as weight (kg) divided by the square of height (m). Physical activity level (PAL) was calculated as total energy expenditure (kcal/day) divided by basal metabolic rate (kcal/day); the former was estimated from the accelerometer as average daily energy expenditure (excluding the initial 3 days), and the latter was defined as basal metabolism standard^25^ × body surface area^26^ × 24 hours. The calculation of energy expenditure by accelerometer has been validated in adults.^27^

### Dietary assessment

Information on diet was collected using a validated short food frequency questionnaire (FFQ) developed by Tokudome et al.^28^^–^^31^ This FFQ was designed to assess the average intakes of 47 of foods and beverage (green tea, coffee, and alcohol) items over the past year. A validation study comparing this FFQ and 3-day weighed diet records revealed that deattenuated, log-transformed, and energy-adjusted Pearson’s correlation coefficients for 26 nutrient intakes ranged from 0.12 to 0.86 (median: 0.45) in males, and from 0.10 to 0.66 (median: 0.38) in females, although mean daily nutrient intakes estimated using the FFQ were generally lower than those estimated from diet records.^29^ Because only 1 item was used to measure alcohol consumption in that FFQ,^28^ this question was removed, and the remaining 46 items were used in this study. Amounts and frequencies were ascertained for 3 staple foods (rice, bread, and noodles) eaten at breakfast, lunch, and supper. The frequency categories (and daily frequencies assigned) for the staple foods were: almost none (0), 1 to 3 times/month (0.1), 1 to 2 times/week (0.2), 3 to 4 times/week (0.5), 5 to 6 times/week (0.8), and daily (1). Each staple food consumed at each meal was converted into bowls/day for rice and noodles or slices (or rolls)/day for bread, and the values obtained for 3 meals were summed. For the other 43 dietary items, only frequency options were given, as follows (assigned daily frequencies in parentheses): almost none (0), 1 to 3 times/month (0.1), 1 to 2 times/week (0.2), 3 to 4 times/week (0.5), 5 to 6 times/week (0.8), once/day (1), twice/day (2), and ≥3 times/day (3).

### Laboratory testing

Parts of the stored serum specimens were sent to an external laboratory (SRL, Hachioji, Japan), and high-sensitivity CRP (hs-CRP) concentrations were measured using a latex-enhanced immunonephelometric assay on a BN II analyzer (Dade Behring, Marburg, Germany). The detection limit of this assay was 50 ng/mL, which was assigned to values below that limit (*n* = 212). Intra- and interassay coefficients of variation for the assay were 3.1% and 1.3%, respectively, at the laboratory.

### Statistical analysis

In the data analysis, we excluded subjects with any of the following conditions: 1) missing data on hs-CRP or dietary habits (*n* = 53), 2) any history of possible inflammation-related disease (cardiovascular disease, cancer, liver disease, or chronic renal failure; *n* = 1552), 3) current use of analgesics (*n* = 328), 4) hs-CRP concentration of 3000 ng/mL or higher (*n* = 592), and 5) dietary energy intake less than 500 or greater than or equal to 3500 kcal/day (*n* = 8). Consequently, 9545 subjects (3905 men and 5640 women) remained for analysis. Among these subjects, some were missing data on alcohol consumption (4 men and 5 women), smoking (1 man and 1 woman), BMI (4 men and 3 women), and PAL (58 men and 56 women).

All analyses were performed separately for men and women with the SAS statistical software package (Ver. 9.1 for Windows; SAS Institute, Cary, NC, USA). Selected major nutrient intakes were estimated by a SAS program utilizing the information from the FFQ and the standard tables of food composition in Japan (fifth revised edition).^32^ The software was kindly provided by the researchers who devised the FFQ.^28^^,^^29^ To identify major dietary patterns based on the 46 food items, factor analysis (equivalent to principal component analysis) of the daily amounts for 3 staple foods and daily frequencies for the other 43 items was performed by using the factor procedure of SAS. The identified factors were rotated by orthogonal transformation (varimax rotation) to improve their interpretability. The natural interpretation of the rotated factors in conjunction with eigenvalues larger than 1 (satisfied for 13 factors in men and 12 factors in women) and the scree plot (ie, a steep decline in the eigenvalue for the next factor; satisfied for 5 factors in men and 4 factors in women) for the corresponding factors before their rotation determined whether a factor should be retained. Accordingly, we evaluated both 4- and 5-factor solutions for each sex, but the 5-factor solution after rotation yielded a more comparable interpretation between men and women. The derived factors (dietary patterns) were labeled based on our interpretation of the data, as well as on the relevant literature. The factor score for each dietary pattern (the dietary pattern score) in an individual was estimated as a linear combination of the standardized values for the food items and the standardized scoring coefficients.

Because the distribution of hs-CRP concentrations was highly skewed to the right, the natural logarithm of hs-CRP was used in all analyses, and the resulting geometric means are presented. In univariate analyses, the *t* test was used to analyze differences in means and the chi-square test was used to evaluate differences in proportions. The associations of each dietary pattern score (categorized into quintiles, which were scored by using the median value of each quintile category) with selected characteristics at baseline were evaluated by linear regression analysis (for continuous variables) or the Mantel test (for categorical variables). Adjusted geometric means of hs-CRP and their 95% confidence intervals by quintiles of each dietary pattern score were computed using the general linear model (GLM) procedure of SAS (analysis of covariance) in 3 different models, to control for potential confounding effects by other factors. The first model was adjusted for age (years) only. The second model was further adjusted for alcohol consumption (never, former, or current drinker consuming 0.1–22.9, 23.0–45.9, or ≥46.0 gramsethanol/day), smoking (never, former, or current smoker of 1–19, 20–39, or ≥40 cigarettes/day), and PAL (continuous). In the third model, BMI was additionally controlled for. Since BMI may represent an intermediate step for possible associations between hs-CRP and dietary patterns, the second and third models were examined separately. The linear trend of the association between hs-CRP and each dietary pattern score was assessed by including in the model a continuous variable with the median value of the score within each quintile category, as well as covariates. A *P* value less than 0.05 was considered statistically significant.

## RESULTS

Table 1 shows the basic characteristics of the study subjects. The mean age was 56 years for men and 55 years for women. Current drinkers accounted for 81% of men and 42% of women, and current smokers represented 37% of men and 8% of women. Men had a higher average BMI than did women (23.7 versus 22.3 kg/m^2^), whereas women had a higher average PAL (1.458 versus 1.467). Men had higher intakes of total energy, carbohydrate, protein, and sodium, and lower intakes of fat, iron, vitamin C, and dietary fiber, than women. The geometric mean of hs-CRP for men was 422 ng/mL, which was significantly higher than that for women (299 ng/mL).

**Table 1.** Characteristics of study subjects

Characteristic	Men (*n* = 3905)	Women (*n* = 5640)	*P*^a^
Age (y)	55.7 ± 8.2^b^	55.1 ± 8.2	<0.01
Alcohol, *n* (%)^c^			
Never drinker	630 (16.2)	3146 (55.8)	<0.01
Former drinker	105 (2.7)	119 (2.1)	
Current drinker	3166 (81.2)	2370 (42.1)	
0.1–22.9 g/d	1399 (35.9)	2112 (37.5)	
23.0–45.9 g/d	898 (23.0)	177 (3.1)	
46.0+ g/d	869 (22.3)	81 (1.4)	
Smoking, *n* (%)^d^			
Never smoker	957 (24.5)	4910 (87.1)	<0.01
Former smoker	1516 (38.8)	267 (4.7)	
Current smoker	1431 (36.7)	462 (8.2)	
1–19 cigarettes/d	407 (10.4)	300 (5.3)	
20–39 cigarettes/d	882 (22.6)	154 (2.7)	
40+ cigarettes/d	142 (3.6)	8 (0.1)	
Body mass index (kg/m^2^)^e^	23.7 ± 3.0	22.3 ± 3.1	<0.01
Physical activity level^f^	1.458 ± 0.09	1.467 ± 0.08	<0.01
Nutrients			
Total energy (kcal/d)	1940 ± 349	1518 ± 228	<0.01
Carbohydrate (g/d)	279 ± 68	211 ± 42	<0.01
Protein (g/d)	56.4 ± 11.0	50.5 ± 9.9	<0.01
Fat (g/d)	42.0 ± 10.6	43.7 ± 10.4	<0.01
Cholesterol (mg/d)	230 ± 63	232 ± 59	0.32
Saturated fat (g/d)	10.7 ± 2.3	11.2 ± 2.5	<0.01
Monounsaturated fat (g/d)	16.0 ± 3.9	16.4 ± 3.8	<0.01
Polyunsaturated fat (g/d)	12.7 ± 3.2	13.1 ± 3.2	<0.01
n-3 polyunsaturated fat (g/d)	2.3 ± 0.6	2.3 ± 0.5	0.66
Sodium (mg/d)	1716 ± 464	1690 ± 444	<0.01
Iron (mg/d)	6.9 ± 1.7	7.3 ± 1.8	<0.01
Vitamin C (mg/d)	87.7 ± 28.0	106.4 ± 34.6	<0.01
Dietary fiber (g/d)	9.6 ± 2.6	11.1 ± 3.1	<0.01
hs-CRP (ng/mL)^g^	422 ± 2	299 ± 3	<0.01

^a^*P* values for sex differences are based on *t* tests for continuous variables and chi-square tests for categorical variables.

^b^Values are mean ± standard deviation for continuous variables and number (percentage) for categorical variables.

^c^Based on 3901 men and 5635 women.

^d^Based on 3904 men and 5639 women.

^e^Based on 3901 men and 5637 women.

^f^Calculated as total energy expenditure (kcal/d) divided by basal metabolic rate (kcal/d) in 3847 men and 5584 women.

^g^Geometric mean.

The results from factor analysis of dietary patterns (ie, factor loading matrix showing correlation coefficients between each dietary pattern identified and 46 food items) are shown in Table 2. We identified 5 major dietary patterns in both sexes. Because of the close similarity in diet between sexes, these patterns were labeled without dividing by sex, as follows: 1) healthy (high in vegetables, fruit, fish, and tofu), 2) Western (high in meat, egg, mayonnaise, and deep- or stir-fried foods), 3) seafood (high in shellfish, squid, octopus, shrimp, crab, fish roe, and fish), 4) bread (high in bread, margarine, and coffee; low in rice and miso soup), and 5) dessert (high in Western/Japanese confections and fruit). These dietary patterns accounted for 12.7%, 6.8%, 5.3%, 4.6%, and 4.1%, respectively, of the variance in food intakes of men, and 11.9%, 6.6%, 5.9%, 4.8%, and 4.1% of the variance in women. Together, they explained 33.5% and 33.3% of the variability in men and women, respectively.

**Table 2.** Factor-loading matrix for the major dietary patterns identified by factor analysis in study subjects^a^

Food item	Healthy		Western		Seafood		Bread		Dessert	
				
	Men	Women	Men	Women	Men	Women	Men	Women	Men	Women
Rice	—^b^	—	—	—	—	—	**−0.68**	**−0.71**	—	—
Bread	—	—	—	—	—	—	**0.68**	**0.72**	0.21	—
Noodles	—	—	—	—	0.28	—	0.31	0.22	−0.20	—
Margarine	—	—	—	—	—	—	**0.52**	**0.56**	—	—
Butter	—	—	—	—	—	—	0.23	—	0.22	—
Milk	—	—	—	—	—	—	0.26	0.27	0.24	—
Yogurt	0.25	—	—	−0.21	—	0.24	0.26	0.28	—	—
Miso soup	0.30	0.25	—	—	—	0.23	**−0.51**	**−0.40**	—	—
Tofu	0.32	0.26	—	—	0.21	0.38	—	—	—	—
Fermented and unfermented soybean	0.32	0.29	—	—	—	**0.42**	—	—	—	—
Eggs	—	—	**0.40**	**0.46**	—	—	—	—	—	—
Chicken	—	—	**0.45**	**0.55**	0.21	0.22	—	—	—	—
Beef/pork	—	—	**0.56**	**0.58**	—	—	—	—	—	—
Liver	—	—	—	0.25	**0.41**	**0.47**	—	—	—	—
Ham/sausage/salami/bacon	—	—	**0.55**	**0.62**	—	—	—	—	—	—
Fish	0.35	0.33	—	—	**0.40**	**0.44**	—	—	—	—
Bone-edible small fish	0.35	0.29	—	—	**0.46**	**0.52**	—	—	—	—
Canned tuna	—	—	0.23	0.34	0.23	0.39	—	—	—	—
Squid/octopus/shrimp/crab	—	—	0.20	0.28	**0.64**	0.29	—	—	—	0.39
Shellfish	—	—	—	—	**0.68**	**0.47**	—	—	—	0.31
Fish roe	—	—	—	0.24	**0.46**	0.32	—	—	—	0.27
Fish paste products	—	—	0.30	0.34	0.33	—	—	—	—	0.32
Tofu products	0.33	0.29	—	—	0.28	0.33	—	—	—	0.21
Potatoes	**0.56**	**0.56**	—	—	—	0.24	—	—	—	—
Pumpkin	**0.53**	**0.51**	—	—	—	0.32	—	—	—	—
Carrots	**0.69**	**0.68**	0.23	—	—	—	—	—	—	—
Broccoli	**0.47**	**0.45**	—	—	—	0.26	—	—	—	—
Green leafy vegetables	**0.67**	**0.71**	—	—	—	—	—	—	—	—
Other green/yellow vegetables	**0.67**	**0.72**	0.21	—	—	—	—	—	—	—
Cabbage	**0.57**	**0.62**	0.30	0.23	—	—	—	—	—	—
Daikon (Japanese radish)	**0.65**	**0.60**	—	—	0.20	0.23	—	—	—	—
Kiriboshi-daikon	0.28	0.30	—	—	0.21	**0.43**	—	—	—	—
Burdock/bamboo shoot	**0.48**	**0.40**	—	—	0.20	0.29	—	—	—	—
Other vegetables	**0.67**	**0.67**	0.33	—	—	—	—	—	—	—
Mushrooms	**0.66**	**0.61**	0.20	—	—	—	—	—	—	—
Seaweed	**0.55**	**0.53**	—	—	—	—	—	—	—	—
Mayonnaise	—	—	**0.58**	**0.54**	—	—	—	—	—	—
Deep-fried foods	—	—	**0.67**	**0.60**	—	—	—	—	—	0.20
Stir-fried foods	0.25	0.32	**0.65**	**0.48**	—	—	—	—	—	—
Citrus fruit	**0.38**	0.36	—	—	—	—	—	—	**0.40**	0.33
Other fruit	**0.46**	**0.45**	—	−0.20	—	—	—	—	0.36	0.33
Peanuts	—	—	—	—	—	—	—	—	0.25	0.32
Western confections	—	—	0.21	0.21	—	—	—	—	**0.70**	**0.60**
Japanese confections	—	—	—	—	—	—	—	—	**0.71**	**0.66**
Green tea	0.20	—	—	—	—	—	−0.24	−0.30	0.20	—
Coffee	—	—	0.30	—	—	—	0.29	**0.40**	—	—

^a^*n* = 3905 for men and *n* = 5640 for women.

^b^For simplicity, factor loadings greater than −0.20 and less than 0.20 are indicated by a dash; those less than or equal to −0.40 or greater than or equal to 0.40 are shown in bold.

Table 3 shows the associations between each dietary pattern score and selected characteristics at baseline. The healthy dietary pattern was positively associated with age and was inversely associated with alcohol (in women only), smoking, and BMI. Conversely, the Western pattern was inversely associated with age and positively associated with alcohol (in women only), smoking, and BMI, as well as PAL. The seafood pattern was characterized by positive associations with age, alcohol (in men only), and BMI and an inverse association with smoking (in women only). The bread pattern was inversely associated with age, alcohol (in men only), BMI (in women only), and PAL (in men only) and positively associated with alcohol (in women only), smoking, and PAL (in women only). The dessert pattern was positively associated with age, BMI (in women only), and PAL (in men only) and inversely associated with alcohol and smoking. Table 3 also shows the associations between each dietary pattern score and estimated intakes of major nutrients.

**Table 3.** Characteristics of study subjects (3905 men and 5670 women) by quintiles (Q) of dietary pattern scores

Characteristic		Healthy			Western			Seafood			Bread			Dessert		
				
		Q1	Q5	*P*^a^	Q1	Q5	*P*^a^	Q1	Q5	*P*^a^	Q1	Q5	*P*^a^	Q1	Q5	*P*^a^
Age (y)	Men	52.9^b^	58.4	<0.01	60.7	51.0	<0.01	54.5	56.3	<0.01	57.8	55.3	<0.01	54.5	56.9	<0.01
	Women	53.0	57.5	<0.01	59.1	51.2	<0.01	52.0	58.8	<0.01	57.9	53.4	<0.01	54.0	56.6	<0.01
Current drinkers (%)^c^	Men	76.7	81.0	0.09	81.5	81.6	0.73	69.5	89.2	<0.01	82.8	79.1	<0.01	90.8	72.3	<0.01
	Women	45.1	36.5	<0.01	37.4	44.9	<0.01	37.9	40.3	0.35	34.8	46.5	<0.01	44.2	39.0	0.02
Current smokers (%)^d^	Men	51.6	24.1	<0.01	25.5	47.9	<0.01	36.6	36.0	0.55	30.2	38.9	<0.01	46.1	29.5	<0.01
	Women	15.0	3.3	<0.01	5.9	10.5	<0.01	10.0	7.2	<0.01	4.0	12.0	<0.01	13.8	4.7	<0.01
Body mass index	Men	23.9	23.5	0.04	23.5	23.8	<0.01	23.5	23.8	0.02	23.7	23.5	0.30	23.6	23.7	0.90
(kg/m^2^)^e^	Women	22.6	22.0	<0.01	22.1	22.4	<0.01	22.0	22.6	<0.01	22.6	21.6	<0.01	22.1	22.5	<0.01
Physical activity level^f^	Men	1.457	1.459	0.51	1.452	1.460	0.04	1.461	1.455	0.13	1.467	1.455	0.03	1.453	1.464	0.01
	Women	1.465	1.466	0.63	1.462	1.473	<0.01	1.466	1.467	0.39	1.465	1.474	0.01	1.468	1.471	0.38
Nutrients																
Total energy (kcal/d)	Men	1885	2017	<0.01	1853	2062	<0.01	1945	2002	<0.01	2167	1840	<0.01	1838	2090	<0.01
	Women	1451	1587	<0.01	1443	1635	<0.01	1517	1570	<0.01	1607	1494	<0.01	1457	1630	<0.01
Carbohydrate (g/d)	Men	272	288	<0.01	268	290	<0.01	288	278	<0.01	332	249	<0.01	247	308	<0.01
	Women	201	222	<0.01	205	221	<0.01	211	218	<0.01	240	195	<0.01	200	228	<0.01
Protein (g/d)	Men	51.4	63.4	<0.01	53.0	62.3	<0.01	53.3	63.6	<0.01	60.5	56.5	<0.01	55.5	60.2	<0.01
	Women	45.7	55.3	<0.01	46.4	57.7	<0.01	47.1	57.5	<0.01	51.7	51.5	0.91	48.9	54.4	<0.01
Fat (g/d)	Men	38.4	47.7	<0.01	34.7	54.1	<0.01	42.3	45.3	<0.01	39.6	47.0	<0.01	40.2	46.9	<0.01
	Women	39.0	48.2	<0.01	36.7	55.6	<0.01	45.5	45.5	0.31	40.6	47.8	<0.01	41.7	48.1	<0.01
Cholesterol (mg/d)	Men	218	254	<0.01	199	270	<0.01	215	262	<0.01	245	224	<0.01	232	245	<0.01
	Women	219	246	<0.01	198	279	<0.01	228	250	<0.01	231	233	0.71	220	257	<0.01
Saturated fat (g/d)	Men	9.9	11.7	<0.01	10.1	11.7	<0.01	10.4	11.3	<0.01	10.1	11.7	<0.01	10.2	11.7	<0.01
	Women	10.5	12.0	<0.01	10.5	12.6	<0.01	10.9	12.0	<0.01	10.4	12.3	<0.01	11.0	12.0	<0.01
Monounsaturated	Men	15.1	17.8	<0.01	12.8	21.4	<0.01	16.2	17.3	<0.01	16.1	16.1	0.82	15.8	17.3	<0.01
​ fat (g/d)	Women	14.8	18.0	<0.01	13.4	21.3	<0.01	17.6	16.6	<0.01	16.5	16.4	0.77	15.5	18.1	<0.01
Polyunsaturated	Men	11.3	14.7	<0.01	10.6	16.3	<0.01	12.6	14.0	<0.01	13.6	12.5	<0.01	12.0	14.1	<0.01
​ fat (g/d)	Women	11.3	14.9	<0.01	11.1	16.4	<0.01	13.5	14.1	<0.01	13.8	13.0	<0.01	12.1	14.7	<0.01
n-3 polyunsaturated	Men	2.1	2.6	<0.01	2.1	2.8	<0.01	2.2	2.6	<0.01	2.4	2.2	<0.01	2.3	2.4	<0.01
​ fat (g/d)	Women	2.0	2.6	<0.01	2.1	2.8	<0.01	2.3	2.5	<0.01	2.4	2.2	<0.01	2.2	2.5	<0.01
Sodium (mg/d)	Men	1472	1978	<0.01	1770	1719	0.24	1619	1936	<0.01	1846	1804	0.54	1678	1818	<0.01
	Women	1476	1885	<0.01	1670	1756	<0.01	1530	1951	<0.01	1786	1713	<0.01	1574	1870	<0.01
Iron (mg/d)	Men	5.6	8.6	<0.01	6.9	7.2	<0.01	6.7	7.6	<0.01	7.9	6.2	<0.01	6.6	7.5	<0.01
	Women	5.9	9.1	<0.01	7.3	7.6	<0.01	6.7	8.7	<0.01	8.1	6.6	<0.01	6.9	8.0	<0.01
Vitamin C (mg/d)	Men	65	120	<0.01	89	92	<0.01	89	93	<0.01	92	87	<0.01	76	105	<0.01
	Women	78	143	<0.01	114	108	<0.01	102	123	<0.01	109	105	<0.01	96	124	<0.01
Dietary fiber (g/d)	Men	7.3	13.0	<0.01	9.8	9.9	<0.01	9.6	10.4	<0.01	9.8	10.2	<0.01	9.1	10.9	<0.01
	Women	8.3	15.0	<0.01	11.7	11.5	0.37	10.7	13.1	<0.01	11.1	11.4	0.03	10.6	12.8	<0.01

^a^*P* values for linear trends across quintiles are based on linear regression analysis for continuous variables and the Mantel test for current drinking and smoking status.

^b^Values are means for continuous variables and percentages for current drinking or smoking status in the first (Q1) and fifth (Q5) quintiles of each dietary pattern score.

^c^Based on 3901 men and 5635 women.

^d^Based on 3904 men and 5639 women.

^e^Based on 3901 men and 5637 women.

^f^Calculated as total energy expenditure (kcal/d) divided by basal metabolic rate (kcal/d) in 3847 men and 5584 women.

The adjusted geometric means of serum hs-CRP and their 95% confidence intervals by quintiles of each dietary pattern score are shown in Table 4. The healthy pattern was significantly associated with lower levels of serum hs-CRP in men and women after adjustment for age, alcohol, smoking, and PAL (*P* for trend <0.01). However, additional adjustment for BMI substantially attenuated this inverse association (*P* = 0.01 in men and *P* = 0.06 in women). After adjustment for all factors except BMI, the Western dietary pattern was significantly positively associated with hs-CRP only in women (*P* < 0.01), but the association was marginal (*P* =0.06) after further adjustment for BMI. The seafood pattern was significantly associated with elevated hs-CRP in men only (*P* = 0.02), even after full adjustment. The bread pattern was inversely associated with hs-CRP in men (*P* = 0.04) and women (*P* < 0.01), without adjusting for BMI; however, after adjustment for BMI, this association became marginal in men (*P* = 0.06) and nonsignificant in women (*P* = 0.33). The dessert pattern was strongly inversely associated with hs-CRP in men only, before and after adjustment for BMI (*P* < 0.01).

**Table 4.** Adjusted geometric means (ng/mL) of serum high-sensitivity C-reactive protein by quintiles (Q) of each dietary pattern score in study subjects

Dietary pattern		Men (*n* = 3905)						Women (*n* = 5640)					
	
		Q1 (lowest)	Q2	Q3	Q4	Q5 (highest)	*P* for trend^a^	Q1 (lowest)	Q2	Q3	Q4	Q5 (highest)	*P* for trend^a^
Healthy	Model 1^b^	472	431	418	405	388	<0.01	315	313	311	284	273	<0.01
		(444–503)^c^	(405–459)	(393–445)	(380–431)	(364–413)		(299–333)	(297–331)	(295–328)	(269–300)	(259–289)	
	Model 2^d^	464	426	418	409	396	<0.01	314	313	312	283	273	<0.01
		(435–494)	(400–453)	(393–445)	(385–436)	(372–422)		(297–331)	(296–330)	(295–329)	(268–299)	(258–288)	
	Model 3^e^	452	423	423	412	402	0.01	296	308	313	289	285	0.06
		(425–480)	(399–449)	(399–448)	(388–437)	(378–426)		(282–312)	(293–324)	(298–329)	(275–304)	(271–300)	
Western	Model 1^b^	398	433	415	419	445	0.07	267	304	292	309	326	<0.01
		(373–425)	(407–461)	(390–441)	(394–446)	(417–475)		(252–282)	(288–321)	(277–309)	(293–326)	(308–344)	
	Model 2^d^	403	434	415	422	437	0.21	266	304	291	309	323	<0.01
		(378–430)	(408–462)	(390–442)	(397–449)	(409–466)		(252–281)	(288–321)	(276–307)	(293–326)	(306–342)	
	Model 3^e^	408	439	419	411	433	0.51	280	311	291	301	309	0.06
		(384–434)	(414–466)	(395–445)	(387–436)	(408–461)		(266–294)	(296–327)	(277–307)	(286–316)	(293–325)	
Seafood	Model 1^b^	387	430	423	428	444	<0.01	300	299	281	305	310	0.31
		(363–412)	(404–458)	(398–451)	(402–455)	(417–472)		(284–317)	(283–315)	(266–297)	(289–322)	(293–327)	
	Model 2^d^	382	431	423	430	448	<0.01	299	302	280	304	307	0.47
		(359–406)	(405–458)	(397–449)	(404–457)	(421–476)		(283–316)	(286–318)	(265–296)	(288–321)	(291–325)	
	Model 3^e^	390	432	420	427	441	0.02	302	302	283	305	300	0.96
		(368–414)	(408–459)	(396–446)	(403–453)	(416–468)		(288–318)	(287–317)	(269–297)	(290–320)	(285–316)	
Bread	Model 1^b^	434	413	429	431	402	0.20	312	307	299	306	272	<0.01
		(408–462)	(388–440)	(403–457)	(405–459)	(378–428)		(295–330)	(291–324)	(283–315)	(290–323)	(257–287)	
	Model 2^d^	447	414	422	431	398	0.04	312	307	298	304	271	<0.01
		(420–476)	(389–440)	(397–449)	(405–459)	(374–423)		(295–330)	(291–324)	(283–315)	(288–321)	(257–286)	
	Model 3^e^	447	413	420	428	403	0.06	303	301	292	304	290	0.33
		(421–474)	(389–438)	(396–446)	(404–454)	(380–427)		(288–319)	(287–317)	(278–307)	(289–320)	(276–305)	
Dessert	Model 1^b^	457	454	407	400	395	<0.01	302	301	291	296	304	0.86
		(430–486)	(427–483)	(382–433)	(376–426)	(371–420)		(286–318)	(286–318)	(276–307)	(280–312)	(288–321)	
	Model 2^d^	450	453	412	400	398	<0.01	300	301	290	295	305	0.76
		(423–479)	(426–482)	(387–438)	(376–425)	(374–424)		(284–317)	(285–318)	(275–306)	(279–311)	(289–322)	
	Model 3^e^	451	450	410	403	400	<0.01	303	303	291	295	300	0.66
		(425–478)	(424–477)	(387–435)	(380–427)	(377–424)		(288–319)	(288–318)	(277–306)	(280–310)	(285–315)	

^a^Based on multiple linear regression analysis; the model included a continuous variable with the median value of dietary pattern score within each quintile category.

^b^Adjusted for age (y).

^c^Geometric mean (95% confidence interval).

^d^Adjusted for age (y), alcohol consumption (never, former, and current drinker consuming 0.1–22.9, 23.0–45.9, and ≥46 g ethanol/d), smoking (never, former, and current smoker of 1–19, 20–39, and ≥40 cigarettes/d), and physical activity level (continuous) in 3842 men and 5579 women.

^e^Adjusted for all variables in Model 2 plus body mass index (kg/m^2^) in 3842 men and 5579 women.

## DISCUSSION

In this study of a middle-aged Japanese population, we identified 5 major dietary patterns—healthy, Western, seafood, bread, and dessert—in both men and women. Most previous studies of diet reported 2 to 4 dietary patterns in 1 sex or both sexes combined.^17^^–^^21^^,^^33^^–^^35^ Analysis of our findings revealed some similarities and some differences with those of previous reports. The healthy pattern (higher intakes of vegetables, fruit, and fish) and Western pattern (higher intakes of meat, eggs, and fried foods) identified in our study are analogous to similarly named patterns used in previous studies of other Japanese populations^21^^,^^33^^–^^35^ and Western populations.^17^^–^^19^ A pattern similar to the bread pattern in the current study was defined as a Westernized breakfast pattern in 2 previous Japanese studies.^21^^,^^36^ One of those studies also observed a seafood pattern similar to the pattern we noted.^21^ The dessert pattern observed in the present study has not been documented in previous studies. We speculate that this pattern might emerge if future studies derive 5 or more dietary patterns, especially in Japanese populations; however, it might be specific to our study population. Further study of this issue is necessary.

This study is one of the largest cross-sectional studies of the association between dietary patterns and CRP and further supports an inverse association between a healthy/prudent dietary pattern and hs-CRP, a finding that has been observed in some,^18^^,^^20^^,^^21^ but not all,^17^^,^^19^^,^^22^ previous studies. High intakes of vegetables and fruit have consistently been used to characterize the healthy/prudent dietary pattern^17^^,^^18^^,^^20^^,^^21^ and have also been associated with lower serum CRP in several studies.^13^^,^^37^^–^^39^ The beneficial combinations of antioxidant vitamins^38^^,^^39^ and fiber^12^ contained in vegetables and fruit may partly mediate this inverse association between healthy dietary pattern and CRP. However, it was evident in our results that this inverse association was partly explained by the association between lower BMI and the healthy dietary pattern. We observed that, after additional adjustment for BMI, the resulting downward trend was not as steep as that reported previously in a Japanese population, particularly in women.^21^

The Western pattern, characterized by high intakes of red meat, refined grains, processed meat, and high-fat dairy products, has consistently been associated with higher CRP concentrations in Western^17^^–^^19^ and Iranian^20^ populations, but not in Japanese^21^ or Alaskan Eskimos.^22^ We observed a positive association between the Western pattern and hs-CRP only in women, and this association was largely mediated by increasing BMI. The Western pattern score was significantly positively associated with estimated fat intake (Table 3), but fat-derived energy intake accounted for a mean of 20% of total energy intake in men as compared with 26% in women (data not shown). Both of these values are substantially lower than corresponding estimates in other studies (eg, 31%–38% in an ethnically diverse US population^19^ and 27%–31% in Iranian women^20^). This may partially explain the absence, or weakening, of an association between hs-CRP and the Western dietary pattern in this study.

Several studies have reported that intakes of fish^14^ and seafood-based long-chain n-3 PUFAs (ie, eicosapentaenoic and docosahexaenoic acids)^40^ are inversely associated with serum CRP concentrations, although these findings have not been consistent.^11^^,^^41^^,^^42^ In the current study, we unexpectedly found that the seafood pattern was positively associated with serum hs-CRP in men, even in the fully adjusted model. A Japanese study evaluated the relation between serum hs-CRP and a seafood dietary pattern (characterized by high intakes of shellfish, salted fish intestines, fish roe, and fish paste), and trends toward positive associations were found (*P* for trend: 0.10 in men and 0.05 in women).^21^ In our male subjects, fish had a lower factor loading (0.40) in the seafood pattern than did other seafood such as shellfish (0.68), squid/octopus/shrimp/crab (0.64), and fish roe (0.46), and estimated intake of n-3 PUFAs was more strongly associated with the healthy or Western pattern than with the seafood pattern (Table 3). The seafood pattern in men was also significantly positively associated with estimated intakes of salt, iron, and cholesterol (Table 3). Higher salt intake might be associated with increased circulating levels of inflammation markers through blood pressure elevation,^43^ and iron intake could lead to higher serum hs-CRP by generating oxidative stress,^44^ although there have been no reports on the association of cholesterol intake and CRP. Because none of these 3 nutrients was positively related to hs-CRP in this study (data not shown), however, we currently have no conclusive explanation for the above finding. The seafood dietary pattern in men was associated with elevated hs-CRP in current drinkers, but not in never or former drinkers (data not shown), and thus we cannot exclude the possibility of residual confounding by alcohol consumption. The factor loadings for the seafood pattern in women differed somewhat from those in men (eg, 0.29 versus 0.64 for squid/octopus/shrimp/crab), which may partly account for the lack of a corresponding association in women.

The bread pattern in the present study can be viewed as the reverse of the traditional Japanese staple food pattern (ie, rice with miso soup and green tea) and was significantly inversely associated with total energy intake as well as carbohydrate intake (Table 3). The observed significant inverse associations between this pattern and hs-CRP became marginal in men and nonsignificant in women after adjusting for BMI, suggesting that a link between decreasing BMI and increasing bread pattern scores largely explains these inverse associations. However, we found that the dessert pattern was strongly associated with lower hs-CRP levels in men, regardless of BMI. The dessert pattern was characterized by high factor loadings for Western/Japanese confections and moderate loadings for citrus and other fruit. Since sucrose and other major nutrients contained in such confections are unlikely to have beneficial effects on serum hs-CRP, some nutrients in fruit (including fruit contained in confectionaries) might be responsible for the above finding. The reason for the absence of an association in women is unclear, but the average daily frequency of fruit consumption was substantially lower in men than in women (0.24 in men versus 0.39 in women for citrus fruit, data not shown), which might have made the effects of the presumed nutrients in fruit evident only in men who could have been deficient in such nutrients. Alternatively, sex differences in factor loadings (eg, moderate loadings for seafood in women) might be relevant.

Some limitations of the present study must be considered. First, due to its cross-sectional design, reverse causation could potentially account for the observed associations. However, we excluded individuals with possible inflammation-related disease or high hs-CRP levels. In addition, most participants did not know their current hs-CRP status, which is not usually tested in health screenings and clinical situations. Because hs-CRP levels in the study subjects were mostly within the normal range, it appears unlikely that elevated or low hs-CRP levels caused a change in their dietary habits. Second, the participation rate of this study was relatively low (about 20%). If subjects with a certain hs-CRP level (eg, low hs-CRP level) who also possessed some dietary habit (eg, high consumption of vegetables and fruit) were more likely to participate for some reason, this could have led to selection bias. However, this possibility also seems unlikely because most subjects were unaware of their hs-CRP status, as described above. Third, our factor analysis was limited in terms of subjectivity in determining and labeling dietary patterns and the difficulty in extrapolating the findings to other populations. Fourth, residual confounding by uncontrolled or unmeasured factors may have distorted true associations with the dietary patterns we identified, which were significantly associated with many important covariates, as shown in Table 3. For example, the use of statins, which could have lowered hs-CRP,^45^ may have confounded those associations, although additional adjustment for hyperlipidemia medication status did not materially alter the current results (data not shown). Thus, our findings need to be confirmed, particularly within other Japanese populations, by well-designed studies that take full account of potential confounders.

In conclusion, we identified 5 major dietary patterns in a Japanese population. Our results indicate that the healthy dietary pattern may be associated with suppressed inflammation, as reflected by decreased hs-CRP, independently of BMI and other factors, in both Japanese men and women. Additional studies are required to assess the possible sex-specific associations of hs-CRP with other dietary patterns (ie, the positive association with the seafood pattern and inverse associations with the bread and dessert patterns in men, and the positive association with the Western pattern in women).
